# Osteosarcoma arising from the parapharyngeal space: A case report

**DOI:** 10.3892/ol.2014.2265

**Published:** 2014-06-18

**Authors:** MANABU HOSHI, JUN TAKADA, NAOTO OEBISU, HIROAKI NAKAMURA

**Affiliations:** Department of Orthopedic Surgery, Osaka City University Graduate School of Medicine, Osaka 545-8585, Japan

**Keywords:** osteosarcoma, parapharyngeal space, head and neck

## Abstract

The current study presents a rare case of osteoblastic osteosarcoma arising from an extremely rare site, namely, the parapharyngeal space. To the best of our knowledge, this is the first study of computed tomography (CT) and magnetic resonance imaging (MRI) of an osteosarcoma in the parapharyngeal space with pathological correlation. A 56-year-old male presented with a mass of the right facial region. CT and MRI showed a heterogeneous mass, with ossification or calcification, occupying the parapharyngeal space. Open biopsy revealed an osteoblastic osteosarcoma containing calcified malignant osteoid. Lung CT also showed multiple lung metastases at the time of the first visit to the Department of Orthopedic Surgery, Osaka City University Graduate School of Medicine (Osaka, Japan). Systemic chemotherapy and radiotherapy were administered to the patient for palliation. The patient was alive at the 24-month follow-up subsequent to this treatment. Although a definitive diagnosis requires the use of a biopsy, the CT and MRI findings described in the present study suggest inclusion of this rare tumor in the differential diagnosis that is formed when such findings occur in the parapharyngeal space. The present study also briefly discusses osteosarcoma of the parapharyngeal space.

## Introduction

Osteosarcoma is rare in the head and neck, accounting for <0.5% (1) of all malignancies of this type. The majority of osteosarcomas in the head and neck are reported to occur in the mandible or maxilla, and few (10%) occur in the skull and facial bones (2). Osteosarcoma arising in the parapharyngeal space is extremely rare. The current study presents this unusual case due to the rarity of the site, and subsequently discusses osteosarcoma of the parapharyngeal space.

## Case report

**Figure 1:**
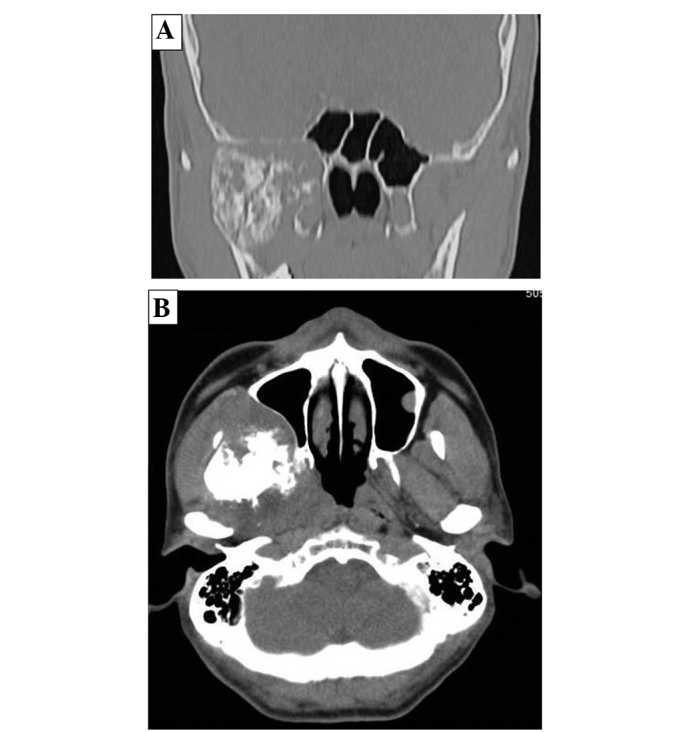
(A) Coronal and (B) axial view of regional computed tomography showing the mass with prominent ossification in the right parapharyngeal space.

**Figure 2:**
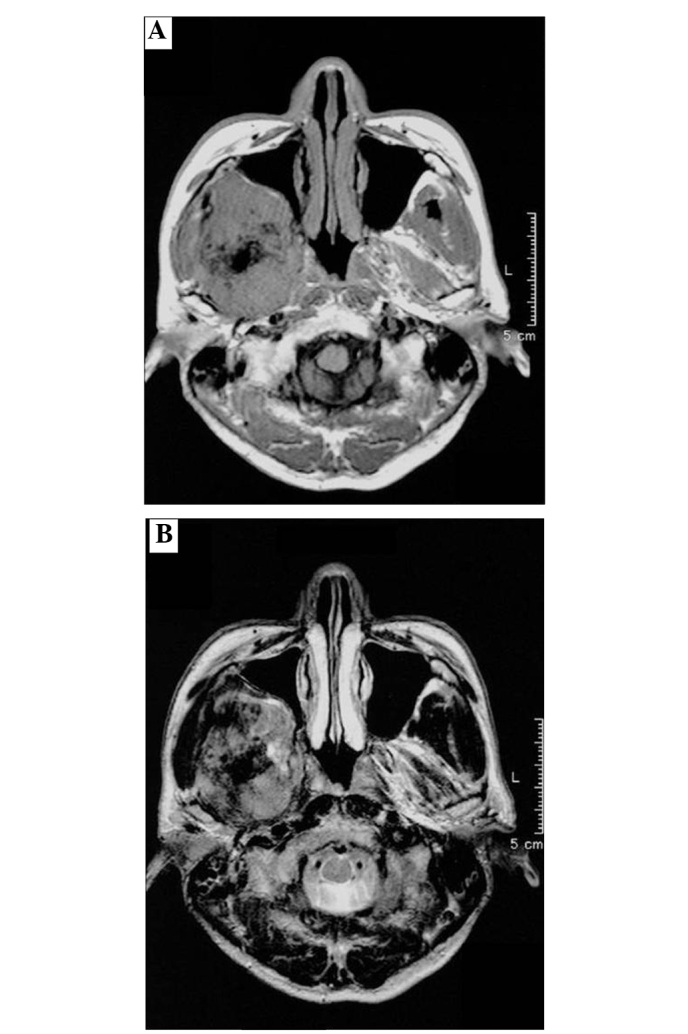
Low-intensity within an isointensity mass on an axial view of magnetic resonance imaging showing ossification in the tumor on (A) T1- and (B) T2-weighted images.

**Figure 3:**
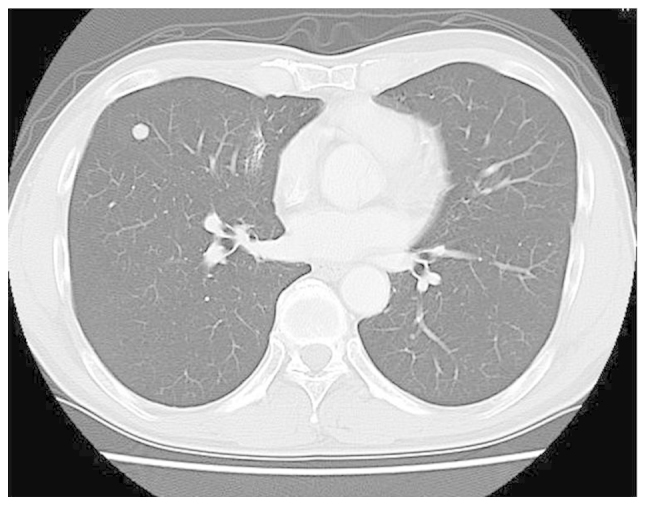
Lung computed tomography showing small nodules of lung metastases.

A 56-year-old male presented with a mass in the right facial bone that had been apparent for three months, and dysesthesia, which had occurred gradually at this site. The patient incidentally identified the tumor and visited Wakakusa-Daiichi Hospital, Higashi-Osaka (Osaka, Japan), where the mass was confirmed with computed tomography (CT) examination. Nothing of note was found in the patient’s past medical and family histories, and the findings from the routine laboratory studies were within the normal limits. Plain X-rays showed no particular findings, but regional CT showed a soft-tissue mass with prominent ossification in the central region, and involving the parapharyngeal space (Fig. 1), in which the anterior septum ballooned owing to compression from the mass. Magnetic resonance imaging (MRI) revealed heterogeneously low intensity to isointensity results on T1-weighted sequences and low to moderately high intensity results on T2-weighted images, with a size of 5.6×4.8 cm (Fig. 2). Lung CT revealed multiple small nodules that were suspected to be lung metastases (Fig. 3).

**Figure 4:**
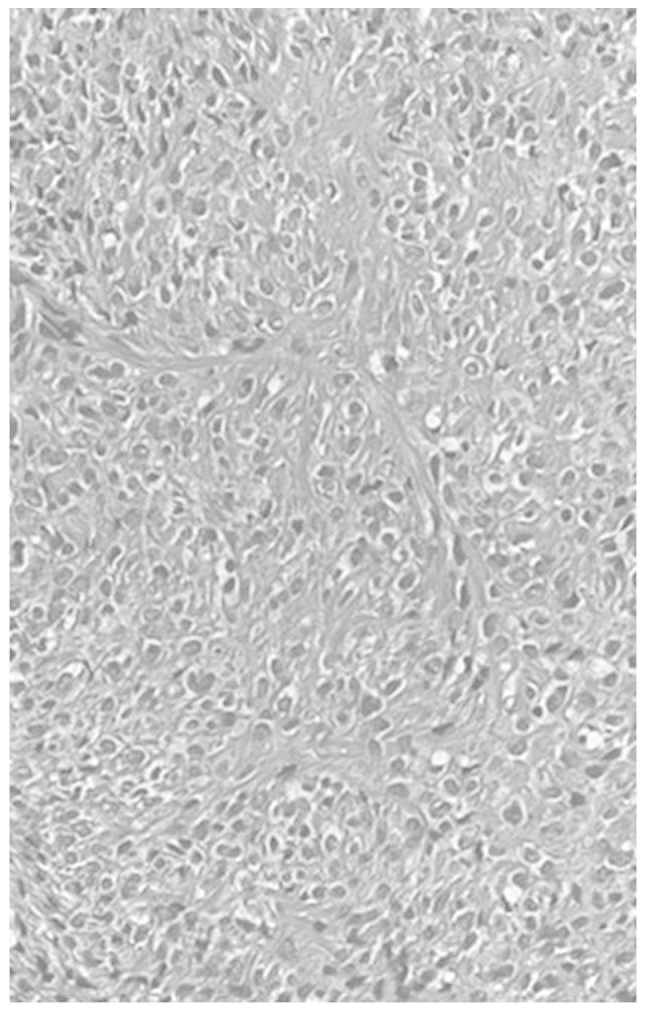
Biopsy specimen confirming osteoblastic osteosarcoma in which atypical cells produce calcified malignant osteoid (stain, hematoxylin and eosin; magnification, ×200).

An open biopsy was performed, and histologically, the lesion showed atypical cell proliferation with production of calcified malignant osteoid. The pathological diagnosis was of an osteoblastic osteosarcoma (Fig. 4). The disease was classified as advanced-stage IVB. At first, systemic chemotherapy was started with caffeine-assisted systemic high-dose chemotherapy, consisting of 60 mg/m^2^ Adriamycin and 120 mg/m^2^ cisplatin. However, subsequent to two courses of the regimen, a perforation of the sigmoid colon was found, and surgical repair was immediately performed with a colostomy. Moreover, irreversible renal dysfunction occurred, and this was worsened by sepsis following surgery. No discernable effect was observed from the chemotherapy on the mass in the parapharyngeal space and the lung target lesion. High-dose chemotherapy was deemed to be unsuitable for treating conventional osteosarcoma owing to the adverse effects. Thus, local radiation therapy, with a total dose of 39 Gy, and a total of seven courses of low-dose systemic chemotherapy (50–100mg/m^2^ cyclophospamide per day) for palliation, were administered. At the last follow-up, 24 months after the first visit, the size of the mass occupying the parapharyngeal space was unchanged, but the size and number of lung metastases had increased. At the time of writing this study, the patient was alive with the disease. Written informed consent was obtained from the patient for publication of this case study and the accompanying images.

## Discussion

Osteosarcoma is a primary high-grade malignant tumor in which the neoplastic cells produce osteoid (3). Osteosarcoma affects males more frequently than females. Young adolescents and adults under the age of 25 years are predominantly affected. Osteosarcoma usually arises in the bones around the knee, humeral joint and pelvis (4). The five-year survival rate is generally estimated to be 60–80% (3). However, the incidence of osteosarcoma in the head and neck regions is extremely low, and this form is reported to occur in adults in their 30s and older (5). Cranial facial lesions account for <10% of the total cases of osteosarcoma; these occur in the mandible and maxillary bones in particular. However it is difficult to find studies on osteosarcoma involving the parapharyngeal space (6).

Radiologically, tumor matrix mineralization and aggressive bone destruction are strongly suggestive of osteosarcoma. The present case demonstrated a high-density mass in the central area of the tumor on CT examination. On MRI, an intermediate intensity region was observed T1-weighted images and a heterogeneously high intensity region was observed on T2-weighted images. An ossified region in the central lesion, which was produced by tumor cells, showed a low intensity on each of the T1- and T2-weighted images. There was no discernible bone-forming reaction to indicate an osteosarcoma (7).

Histologically, osteosarcoma is an osteoid-producing tumor, and the identification of anaplastic stromal cells and the osteoid they produce aid in the histological diagnosis. The incidence of the classic type of osteosarcoma presents as osteoblastic (50%), chondroblastic (25%), or fibroblastic (25%) in the extremities (3). Among head and neck osteosarcomas, chondroblastic osteosarcoma is the most common histopathological variant (8), while osteoblastic osteosarcoma is associated with a poor prognosis, differing from the chondroblastic type in the extremities (5).

Osteosarcoma usually presents with an aggressive course, with a high rate of distant metastasis and recurrence. In the absence of distant metastasis, the major therapeutic approach is surgery to achieve local control for high-grade osteosarcoma; surgery with a negative margin is necessary (9), and multimodality management, in addition to chemotherapy (10) and radiotherapy (11), is preferable. The anatomical site of the parapharyngeal space occupied by the tumor is adjacent to more critical structures in the head and neck, and radical surgery would have great disadvantages in terms of function and possible cosmetic problems. However, systemic chemotherapy with high-dose treatment may be the main treatment for cases with distant metastases. The adverse effect of irreversible renal dysfunction emerged in the present case following the two initial courses of chemotherapy, and high-dose chemotherapy could not be recommended.

The five-year survival rate of patients with head and neck osteosarcoma is estimated to be 57–63% (2,11), which is lower than the 70–80% survival rate of patients with osteosarcoma of the extremities (12). Regarding prognostic factors, Smith *et al* (5) proposed that a poor prognosis is associated with an age of >60 years, a non-mandibular tumor location, a tumor size of >6 cm, an osteoblastic histological type, an advanced disease stage, non-surgical initial therapy and a positive margin of resection. At the latest follow-up, the present patient was alive with the disease, but unfortunately possessed a number of these poor prognostic factors.

In conclusion, the present study presents a rare case of osteoblastic osteosarcoma arising from a rare lesion of the parapharyngeal space. Although a definitive diagnosis requires a biopsy, the CT and MRI findings described in this study suggest the inclusion of this rare tumor in the differential diagnosis when such findings occur in the parapharyngeal space.
